# Association between hypertensive pregnancy disorders and future risk of stroke in Taiwan: a Nationwide population-based retrospective case-control study

**DOI:** 10.1186/s12884-020-02898-9

**Published:** 2020-04-15

**Authors:** Chun-Chung Huang, Chien-Chu Huang, Shao-Yi Lin, Cherry Yin-Yi Chang, Wu-Chou Lin, Chi-Hsiang Chung, Fu-Huang Lin, Chang-Huei Tsao, Chun-Min Lo, Wu-Chien Chien

**Affiliations:** 1 Department of Biomedical Engineering, National Yang-Ming University, 2, Linong St., Beitou Dist, Taipei City, 112 Taiwan; 2 Graduate Institution of Biomedical Sciences, China Medical University, No. 2, Yuh-Der Road, Taichung, 404 Taiwan; 3 Department of Obstetrics and Gynecology, China Medical University Hospital, Taichung, Taiwan; 4 Department of Mechanical and Computer-Aided Engineering, National Formosa University, No. 64, Wunhua Rd, Huwei Township, Yunlin County 632 Taiwan; 5 Department of Medicine, China Medical University, No. 2, Yuh-Der Road, Taichung, 404 Taiwan; 6 School of Public Health, National Defense Medical Center, No. 325, Section 2, Cheng-Kung Road, Neihu District, Taipei City, 11490 Taiwan; 7 Taiwanese Injury Prevention and Safety Promotion Association (TIPSPA), No. 325, Section 2, Cheng-Kung Road, Neihu District, Taipei City, 11490 Taiwan; 8 Department of Medical Research, Tri-Service General Hospital, National Defense Medical Center, No.325, Section 2, Cheng-Kung Road, Neihu District, Taipei City, 11490 Taiwan; 9 Department of Microbiology & Immunology, National Defense Medical Center, No. 325, Section 2, Cheng-Kung Road, Neihu District, Taipei City, 11490 Taiwan; 10 Graduate Institute of Life Sciences, National Defense Medical Center, Taipei City, Taiwan; 11 School of Public Health, National Defense Medical Center, Taipei City, Taiwan

**Keywords:** Gestational hypertension, Pre-eclampsia, Eclampsia, Cerebrovascular disease, CVA, Stroke

## Abstract

**Background:**

The incidence of female stroke has increased gradually and has begun occurring at a younger age in recent years. Given that women live longer than men, stroke would cause more negative and longer-term impacts on the rest of the lives of women. There are few related studies on Asian women. We aimed to evaluate stroke risk in Asian women following hypertensive pregnancy disorders.

**Methods:**

Using the Taiwan National Health Insurance database, we designed a retrospective study that included pregnant women between 2000 and 2013. We selected an age-matched control group of women without hypertensive pregnancy disorders at a 1:3 ratio. The endpoint was any episode of stroke; otherwise, the patients were tracked until December 31, 2013. After the index date until the end of 2013, Cox proportional hazards analysis was used to compare the risk of incident stroke. The risk factors for stroke were determined using Cox proportional regression to calculate the hazard ratio (HR) compared with the control group.

**Results:**

During the follow-up period, the Kaplan-Meier analysis indicated that patients with hypertensive pregnancy disorders had a significantly higher risk of developing stroke than did patients without hypertensive pregnancy disorders (log-rank test *P <* 0.001). Multivariate Cox regression analysis demonstrated that the case group had a 2.134-fold increased risk of stroke (HR = 2.134; 95% CI = 1.817–2.505; *P <* 0.001).

**Conclusion:**

Our study provided evidence of an increased risk of stroke in patients with hypertensive pregnancy disorders. Compared with those without such disorders, the patients who had experienced the disorders had a 2.134-fold (*P* < 0.001) higher risk of developing stroke in the future.

## Background

Hypertensive pregnancy disorders are a set of pregnancy-specific systemic diseases with a unique pathophysiology [1–5]. There are three main types of the disease: gestational hypertension, pre-eclampsia-eclampsia, and superimposed pre-eclampsia. Preeclampsia is a multisystem disorder accompanied by the new onset of hypertension and end-organ dysfunction with or without proteinuria. The critical conditions of hypertensive pregnancy disorders include eclampsia, hemolysis, elevated liver enzymes, low platelet counts, and disseminated intravascular coagulopathy [6–9]. Hypertensive pregnancy disorders are critical risk factors for pregnancy-associated stroke [10]. They affect approximately 5% of pregnant women and increase the risk of pregnancy-associated cerebrovascular disease during the intrapartum and postpartum periods [11–14]. Cerebrovascular dysfunction related to hypertensive pregnancy disorders can lead to stroke, cerebral edema, seizures, and maternal mortality [10, 15, 16].

A recent study revealed that although women were more likely to survive after stroke, poorer recovery was noted [17]. However, most women did not know they were facing the risk of stroke. According to the JAMA guidelines, an estimated 425,325 new or recurrent strokes occur in women in the United States each year, representing approximately 53.5% of the stroke population. In 2010, women accounted for approximately 60% of stroke-related deaths (77,109 of 129,476 deaths). Approximately 3.8 million women live after having a stroke in the United States [18]. Stroke is also the third-leading cause of death in Taiwan, and the mortality rate of stroke was 7.2% in 2012 [19]. Based on the 1994 National Health Interview Survey, women accounted for approximately 46% of the stroke population in Taiwan [20]. The related risk factors for stroke included hypertension, diabetes, hyperlipidemia, obesity, atrial fibrillation, and smoking [21–25].

However, few studies have mentioned the association between stroke not occurring during pregnancy or the postpartum period and hypertensive pregnancy disorders [26–29]. Thus, our study determined the risk of future stroke in women in Taiwan with hypertensive pregnancy disorders.

## Methods

### Data sources

The National Health Insurance program was implemented in 1995 in Taiwan, and the National Health Insurance Research Database (NHIRD) records the medical information of all insured people. The database includes approximately 23.74 million people in Taiwan, and it obtained a coverage rate of approximately 99.6% in 2009 [30, 31]. For study purposes, from 1 million individuals randomly selected as subjects obtained from the NHIRD, we created a small database. The International Classification of Diseases, Ninth Revision, Clinical Modification (ICD-9-CM) was used for the diagnostic and treatment codes in the NHIRD. With the permission of the National Health Research Institute, our study was able to use the databank. This study was granted approval by the Institutional Review Board of Tri-Service General Hospital (TSGHIRB No. 1–105–05-142).

### Study design and sampled participants

**Fig. 1:**
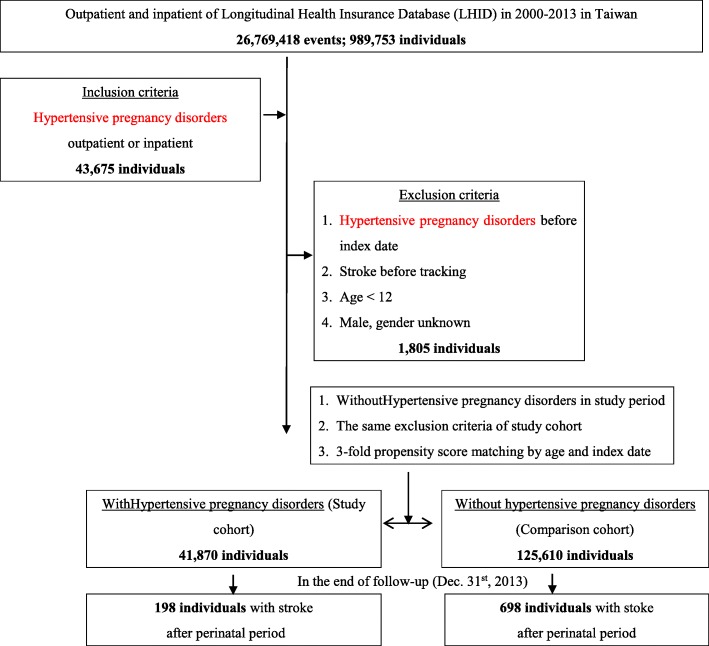
The flowchart of study sample selection from the National Health Insurance Research Database in Taiwan

This study was conducted with a retrospective case-control design with outpatient and inpatient data. We collected our data from January 1, 2000 to December 31, 2013. Among the 989,753 individuals, 43,675 individuals were diagnosed with hypertensive pregnancy disorders (ICD-9 codes 642.0 to 642.9) prior to the index date. The patients were excluded if they met one of the following criteria: diagnosed with hypertensive pregnancy disorders before the index date, diagnosed with one stroke episode before tracking, aged < 12 years, and male sex. At the end of the study, the case group comprised 41,870 individuals. A control group matched by index day and age with propensity scores was enrolled at a 3:1 ratio. The same exclusion criteria as that used for the case group was implemented, and the controls did not have hypertensive pregnancy disorder episodes in the study period; 125,610 individuals comprised the control group. The tracking event refers to the occurrence of stroke, and tracking continued until December 31, 2013. Individuals with any diagnoses of cerebrovascular disease (ICD-9 codes: 436.0 to 436.9 and 437.0 to 437.9) were defined as being diagnosed with stroke (Fig. 1).

### Outcome measures

All study participants were followed from the index date until the onset of stroke (ICD-9 codes: 436.0 to 436.9 and 437.0 to 437.9), withdrawal from the insurance program, or the end of 2013. We eliminated peripartum strokes and postpartum strokes within 3 months (Table S3).

### Covariates

The covariates were age group, area of residence, urbanization level of residence and annual income. The age group was separated into 12–19, 20–29, 30–39, and > 40 years. The area of residence was divided into northern, central, southern, and eastern regions, as well as surrounding islands. The urbanization level of residence was defined based on multiple indicators of development. Level 1 was defined as a population > 1,250,000, with more political, economic, cultural and metropolitan development. Level 2 was a population between 500,000 and 1,249,999. Level 4 was populations less than 149,999, and level 3 was in between levels 2 and 4. The annual income in the United States Dollar (USD) was separated into three class intervals: < 7200, 7200 to 13,999, and ≥ 14,000. Regarding the related comorbidities, we included hyperlipidemia (ICD-9 code: 272), diabetes mellitus (DM) (ICD-9 code: 250), heart disease (ICD-9 codes: 410–429), hypertension (HTN) (ICD-9 codes: 401–405), chronic kidney disease (CKD) (ICD-9 codes: 274.1, 403–404, 440.1, 442.1, 447.3, 572.4, 580–589, 642.1, and 646.2) and obesity (ICD-9 code: 278).

### Statistical analysis

We conducted the analyses using SPSS 20 software (SPSS, Inc., Chicago, IL, USA). Descriptive statistics were used for basic information, including percentages, average values, and standard deviations.

Differences in the distribution of age, insurance premium, sex, season, location, urbanization level, comorbidities, and level of hospital between the two groups and between subjects with and without stroke were compared using the chi-square test. The Cox proportional hazards regression model was also applied to assess the influence of hypertensive pregnancy disorders on the risk of stroke. The relevant hazard ratios (HRs) and 95% confidence intervals (CIs) are presented. The difference in stroke risk between the two groups was estimated by the Kaplan-Meier method with the log-rank test. All results were statistically significant if the two-tailed *p* value was less than 0.05.

## Results

**Fig. 2:**
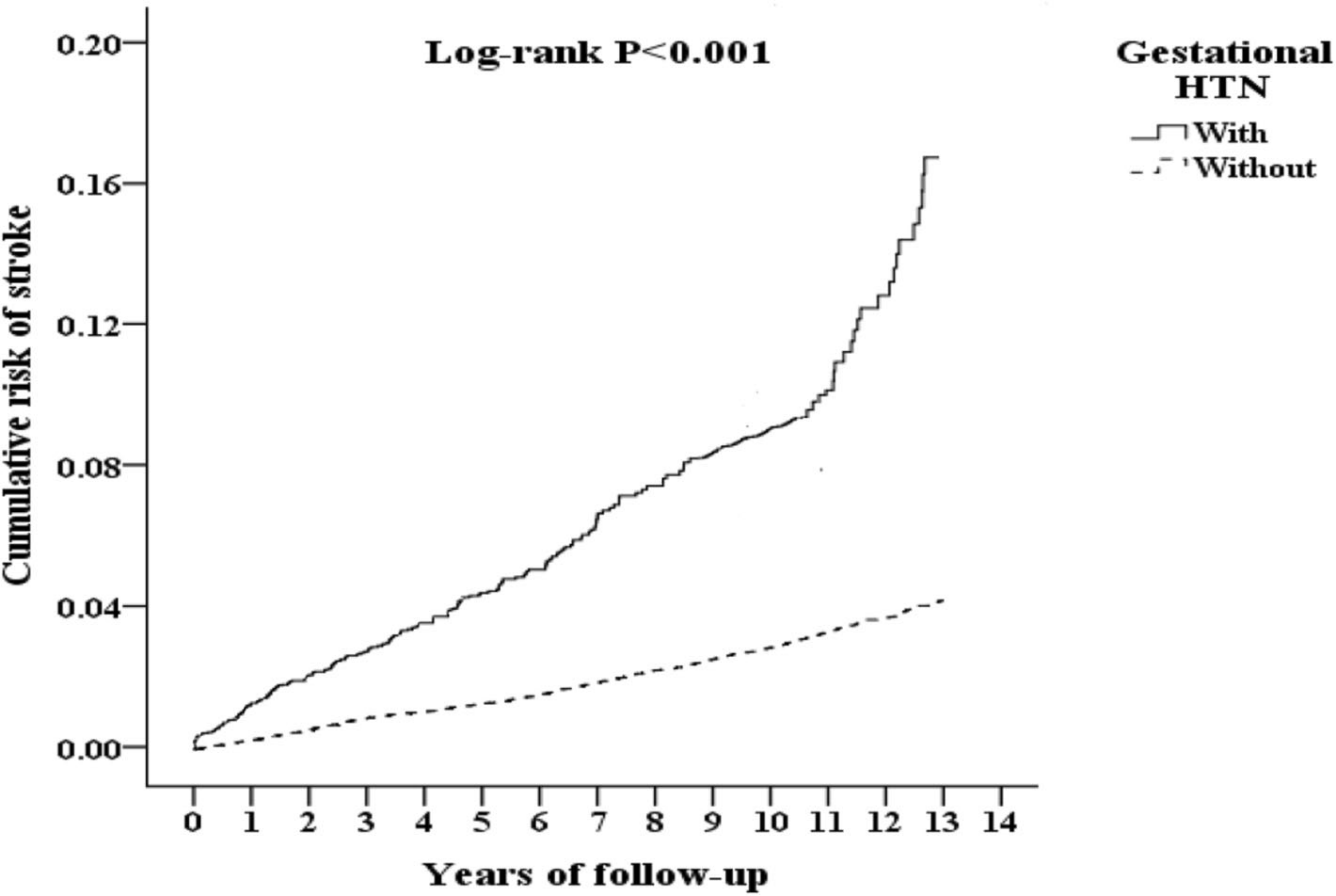
Kaplan-Meier assessment of cumulative risk of stroke among females aged 12 and over stratified by hypertensive pregnancy disorders with log-rank test

This study examined 41,870 patients with hypertensive pregnancy disorders and 125,610 controls. At the 13-year follow-up, the cumulative incidence of stroke was 0.47% (198/41,870 individuals) in patients with hypertensive pregnancy disorders and 0.56% (698/125,610 individuals) in patients without hypertensive pregnancy disorders (Fig. 1 and Table S1). The Kaplan-Meier analysis indicated that patients with hypertensive pregnancy disorders had a significantly higher risk of developing stroke than did patients without hypertensive pregnancy disorders (log-rank test *P <* 0.001) (Fig. 2). Strokes that occurred during the pregnancy and within 3 months postpartum were eliminated. These strokes were only a small portion of the total burden of disease (Table S3).

In the 13th year of follow-up, the case group was more likely to develop stroke than the control group (*p* < 0.001). At the end of follow-up, compared to the controls, patients with hypertensive pregnancy disorders tended to be younger in age (35.49 years old vs. 36.71 years old; *p* < 0.001), have lower insurance premiums (95.48% vs. 94.77%; *p* < 0.001) and have higher rates of diabetes mellitus (2.78% vs. 1.42%; *p* < 0.001), HTN (3.29% vs. 1.37; *p* < 0.001), and obesity (0.14% vs. 0.06; *p* = 0.001). Compared to the controls, more patients with hypertensive pregnancy disorders were treated in the hospital center (32.72% vs. 26.89%) and regional hospital (37.65% vs. 32.53%) (*P* < 0.001) (Table 1).

**Table 1:** Characteristics of the study population in regard to the endpoint

Hypertensive pregnancy disorders	Total		With		Without		***P***
Variables	n	%	n	%	n	%	
**Total**	167,480		41,870	25.00	125,610	75.00	
**Stroke subgroup**							< 0.001
Without	166,584	99.47	41,672	99.53	124,912	99.44	
Acute, but ill-defined, cerebrovascular disease	201	0.12	80	0.19	121	0.10	
Other and ill-defined cerebrovascular disease	695	0.41	118	0.28	577	0.46	
**Age (years)**	36.41 ± 7.07		35.49 ± 6.44		36.71 ± 7.28		< 0.001
**Age group (years)**							< 0.001
12–19	1645	0.98	418	1.00	1227	0.98	
20–29	48,363	28.88	13,191	31.50	35,172	28.00	
30–39	90,913	54.28	22,212	53.05	68,701	54.69	
40–49	24,790	14.80	5666	13.53	19,124	15.22	
≧50	1769	1.06	383	0.91	1386	1.10	
**Insurance premium (USD)**							< 0.001
< 7200	159,022	94.95	39,979	95.48	119,043	94.77	
7200-13,999	7222	4.31	1511	3.61	5711	4.55	
≥ 14,000	1236	0.74	380	0.91	856	0.68	
**DM**	2950	1.76	1165	2.78	1785	1.42	< 0.001
**HTN**	3096	1.85	1379	3.29	1717	1.37	< 0.001
**Hyperlipidemia**	893	0.53	312	0.75	581	0.46	0.224
**Obesity**	129	0.08	59	0.14	70	0.06	0.001
**Heart disease**	2708	1.62	684	1.63	2024	1.61	0.892
**CKD**	1394	0.83	377	0.90	1017	0.81	0.794
**Season**							< 0.001
Spring	36,206	21.62	9454	22.58	26,752	21.30	
Summer	47,389	28.30	10,265	24.52	37,124	29.55	
Autumn	41,478	24.77	11,104	26.52	30,374	24.18	
Winter	42,407	25.32	11,047	26.38	31,360	24.97	
**Location**							< 0.001
Northern Taiwan	67,388	40.24	17,254	41.21	50,134	39.91	
Middle Taiwan	43,559	26.01	10,134	24.20	33,425	26.61	
Southern Taiwan	40,128	23.96	10,004	23.89	30,124	23.98	
Eastern Taiwan	15,413	9.20	4101	9.79	11,312	9.01	
Surrounding islands	992	0.59	377	0.90	615	0.49	
**Urbanization level**							< 0.001
1 (The highest)	63,431	37.87	15,297	36.53	48,134	38.32	
2	75,770	45.24	19,784	47.25	55,986	44.57	
3	10,955	6.54	2111	5.04	8844	7.04	
4 (The lowest)	17,324	10.34	4678	11.17	12,646	10.07	
**Level of care**							< 0.001
Hospital center	47,476	28.35	13,701	32.72	33,775	26.89	
Regional hospital	56,621	33.81	15,765	37.65	40,856	32.53	
Local hospital	63,383	37.85	12,404	29.63	50,979	40.59	

*P:* Chi-square/Fisher exact test for categorical variables and t-test for continuous variables

The Cox regression analysis of the factors associated with the risk of stroke showed that the crude HR was 2.704 (95% CI = 2.326–3.144, *P <* 0.001). After adjusting for the season, the urbanization level of residence and monthly income, the adjusted HR was 2.134 (95% CI = 1.817–2.505, *P <* 0.001). Compared with younger women (aged 12–19 years old), for women between 20 and 29 and 30–39 years old, the adjusted HRs were 1.306 (*P* < 0.001) and 1.215 (*P* = 0.011), respectively. Our results indicated that patients with hypertensive pregnancy disorders had a 2.134-fold higher risk of developing stroke than did individuals without hypertensive pregnancy disorders (Table 2 and S2). Those with diabetes mellitus (DM) (*P* < 0.001), hypertension (HTN) (*P* < 0.001), hyperlipidemia (*P* < 0.001), heart disease (*P* < 0.001), and chronic kidney disease (CKD) (*P* < 0.001) had a higher risk of developing stroke than those without these comorbidities. A higher incidence of stroke development was observed among hypertensive pregnancy disorder patients who visited the hospital center than among those who visited the local hospital (Table 2).

**Table 2:** Factors associated with stroke according to Cox regression

Variables	Crude HR	95% CI	95% CI	***P***	Adjusted HR	95% CI	95% CI	***P***
Hypertensive pregnancy disorders								
Without	Reference				Reference			
With	2.704	2.326	3.144	< 0.001	2.134	1.817	2.505	< 0.001
**Age group (years)**								
12–19	Reference				Reference			
20–29	1.105	1.048	1.261	0.029	1.306	1.137	1.942	< 0.001
30–39	1.129	1.060	1.278	0.024	1.215	1.087	1.320	0.011
40–49	1.094	0.452	1.413	0.367	1.285	0.913	1.248	0.218
≧50	0.341	0.158	1.012	0.055	0.537	0.084	1.065	0.066
**Insured premium (USD)**								
< 7200	Reference				Reference			
7200-13,999	1.458	0.149	2.271	0.224	1.966	0.522	2.582	0.249
≥ 14,000	1.755	0.447	2.425	0.124	2.650	0.157	4.762	0.179
**DM**	4.190	3.532	4.970	< 0.001	1.449	1.277	1.874	< 0.001
**HTN**	7.696	6.742	8.903	< 0.001	4.274	3.616	5.050	< 0.001
**Hyperlipidemia**	8.175	6.559	10.177	< 0.001	3.061	2.415	3.886	< 0.001
**Obesity**	2.607	0.910	6.490	0.067	1.115	0.418	2.995	0.584
**Heart disease**	4.027	3.353	4.837	< 0.001	2.136	1.761	2.589	< 0.001
**CKD**	4.444	3.466	5.699	< 0.001	2.356	1.829	3.041	< 0.001
4 (The lowest)	Reference				Reference			
**Level of care**								
Hospital center	1.876	1.592	2.213	< 0.001	1.631	1.349	1.972	< 0.001
Regional hospital	1.209	1.020	1.436	0.011	2.133	0.885	1.251	0.242
Local hospital	Reference				Reference			

*HR* hazard ratio, *CI* confidence interval, Adjusted HR: Adjusted for variables listed in the table

Adjusted variables: geographical area of residence, urbanization level of residence, and season

**Fig. 3:**
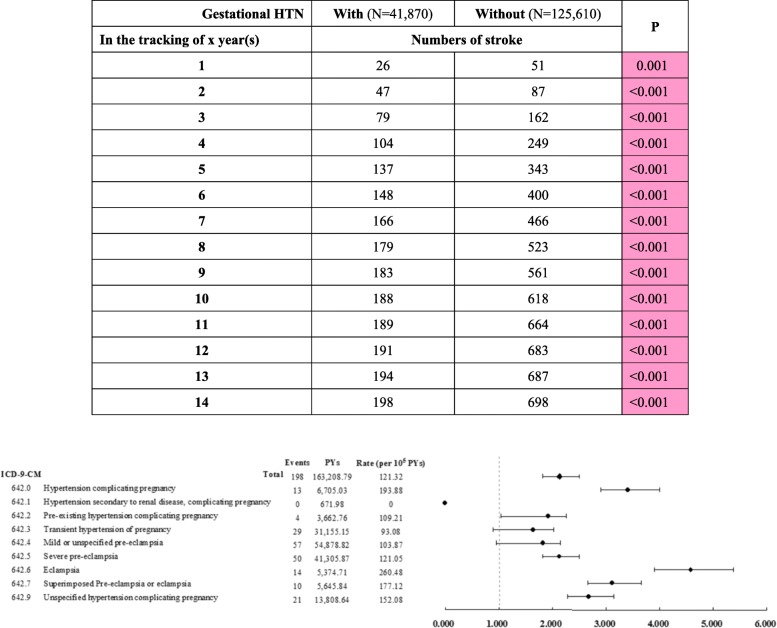
Factors of stroke stratified by hypertensive pregnancy disorder subgroup using Cox regression

The incidence and HR of stroke in populations with or without hypertensive pregnancy disorders relative to those in controls are listed in Table 3. After adjusting for all the other variables, regardless of the other factors, compared with patients without hypertensive pregnancy disorders, those with hypertensive pregnancy disorders had a rate of 1.21 per 1000 patient-years vs. 1.06 per 1000 patient-years in those without gestational hypertension and an HR of stroke that was 2.134-fold (*P* < 0.001) higher than that of those without gestational hypertension (Table 3 and Fig. 3).

**Table 3:** Factors associated with stroke stratified by variables listed in the table by using Cox regression

Hypertensive pregnancy disorders	With			Without *(Reference)*			Ratio	Adjusted HR	95% CI	95% CI	***P***
Stratified	Events	PYs	Rate (per 10^**5**^ PYs)	Events	PYs	Rate (per 10^**5**^ PYs)					
**Total**	198	163,208.79	121.32	698	654,982.30	106.57	1.138	2.134	1.817	2.505	< 0.001
**Age group (years)**											
12–19	2	271.80	735.83	3	589.64	508.78	1.446	2.711	2.308	3.182	< 0.001
20–29	64	32,914.98	194.44	87	80,370.21	108.25	1.796	3.367	2.867	3.953	< 0.001
30–39	97	77,048.17	125.90	303	238,213.86	127.20	0.990	1.855	1.580	2.178	< 0.001
40–49	34	18,003.53	188.85	294	137,974.59	213.08	0.886	1.661	1.415	1.950	< 0.001
≧50	1	34,970.30	2.86	11	197,833.99	5.56	0.514	0.964	0.821	1.132	0.276
**Insured premium (USD)**											
< 7200	191	125,404.75	152.31	688	466,665.85	147.43	1.033	1.937	1.649	2.273	< 0.001
7200-13,999	5	2344.91	213.23	8	8975.42	89.13	2.392	4.484	3.818	5.264	< 0.001
≥ 14,000	2	35,459.13	5.64	2	179,341.03	1.12	5.058	9.481	8.073	11.129	< 0.001
**DM**	20	8430.94	237.22	56	42,672.99	131.23	1.808	3.389	2.885	3.978	< 0.001
**HTN**	54	13,688.23	394.50	99	35,172.46	281.47	1.402	2.627	2.237	3.084	< 0.001
**Hyperlipidemia**	13	1980.06	656.55	42	8226.33	510.56	1.286	2.411	2.052	2.830	< 0.001
**Obesity**	1	477.68	209.35	1	2343.45	42.67	4.906	9.196	7.830	10.795	< 0.001
**Heart disease**	14	5039.75	277.79	75	33,325.22	225.05	1.234	2.314	1.970	2.716	< 0.001
**CKD**	6	2500.94	239.91	40	23,770.74	168.27	1.426	2.673	2.276	3.137	< 0.001
**Season**											
Spring	52	30,039.53	173.11	146	109,482.26	133.35	1.298	2.433	2.072	2.856	< 0.001
Summer	40	32,853.28	121.75	175	126,710.16	138.11	0.882	1.653	1.407	1.940	< 0.001
Autumn	61	37,155.10	164.18	191	132,193.49	144.49	1.136	2.130	1.814	2.500	< 0.001
Winter	45	63,160.87	71.25	186	286,596.39	64.90	1.098	2.058	1.752	2.416	< 0.001
**Urbanization level**											
1 (The highest)	75	43,701.39	171.62	225	153,595.67	146.49	1.172	2.196	1.870	2.578	< 0.001
2	83	57,996.46	143.11	294	210,128.07	139.91	1.023	1.917	1.633	2.251	< 0.001
3	16	10,060.20	159.04	40	46,748.29	85.56	1.859	3.484	2.967	4.090	< 0.001
4 (The lowest)	24	51,450.75	46.65	139	244,510.27	56.85	0.821	1.538	1.310	1.806	< 0.001
**Level of care**											
Hospital center	96	43,471.31	220.84	301	142,056.49	211.89	1.042	1.954	1.663	2.293	< 0.001
Regional hospital	64	50,293.76	127.25	215	183,114.94	117.41	1.084	2.032	1.730	2.385	< 0.001
Local hospital	38	69,443.72	54.72	182	329,810.87	55.18	0.992	1.859	1.583	2.182	< 0.001

*PYs* Person-years; *Adjusted HR* Adjusted hazard ratio: Adjusted for the variables listed in Table 3; *CI* confidence interval

When we analyzed factors related to stroke and stratified patients by hypertensive pregnancy disorder subgroup using Cox regression, the risk of stroke was 3.412-fold (95% CI = 2.905–4.005) higher in patients with a benign essential hypertension-complicated pregnancy (ICD-9: 642.0) (*P* < 0.001), and the rate was 1.9388 per 1000 patient-years. The risk of stroke was 2.130-fold (95% CI = 1.814–2.500) higher in patients with severe preeclampsia (ICD-9: 642.5) (*P* < 0.001), and the rate was 1.2105 per 1000 patient-years. The risk of stroke was 4.584-fold (95% CI = 3.903–5.380) higher in patients with eclampsia (ICD-9: 642.6) (*P* < 0.001), and the rate was 2.6048 per 1000 patient-years. The risk of stroke was 3.117-fold (95% CI = 2.654–3.659) higher in patients with pre-eclampsia or eclampsia superimposed on pre-existing hypertension (ICD-9: 642.7) (*P* < 0.001), and the rate was 1.7712 per 1000 patient-years. The risk of stroke was 2.676-fold (95% CI = 2.279–3.141) higher in patients with a hypertension-complicated pregnancy (ICD-9: 642.9) (*P* < 0.001), and the rate was 1.5208 per 1000 patient-years (Fig. 3).

## Discussion

Hypertensive pregnancy disorders are the most common cause of hypertension in pregnant women [5]. They are classified into 3 categories: gestational hypertension, preeclampsia-eclampsia, and superimposed preeclampsia [32]. Women with chronic high blood pressure experience more complications during pregnancy [12]. High blood pressure may also lead to other long-term health problems after pregnancy. Stroke is an undesirable condition among younger women, as it is considered to have adverse effects on personal status, families and society. Health issues not only affect women but also demand major changes of the whole family. The incidences of both early- and late-onset preeclampsia increased in Taiwan from 2001 to 2014, especially for early-onset disease [33]. Preeclampsia has become an important issue to be aware of.

Studies show that hypertensive pregnancy disorders not only affect the pregnancy period but also increase the risk of cardiovascular disease later in life [34, 35]. Pregnancy-related hypertension is thought to be an important risk factor for both cerebrovascular disease and intracranial venous thrombosis in some studies [36]. One study based on the general population during the intrapartum and postpartum periods in Taiwan revealed that preeclampsia/eclampsia are the two most common causes of intracranial hemorrhage and the three most common causes of cerebral infarction during the intrapartum and postpartum periods [37].

In one cohort study in Sweden, the authors found that an increased risk of cardiovascular disease and cerebrovascular disease after hypertensive disease of pregnancy persisted in the older population [21]. One study in Taiwan previously revealed that the respective adjusted relative risk of hemorrhagic and ischemic cerebrovascular disease after preeclampsia-eclampsia was much higher, regardless of whether it was within the first 3 days postpartum or within 1 year of the postpartum period [38]. One retrospective cohort study based on the Utah Population Database also showed that women with hypertensive disease of pregnancy have increased mortality risk, particularly with respect to ischemic heart disease and cerebrovascular disease [39].

Our study revealed an important issue in hypertensive pregnancy disorders during pregnancy. Strokes that did not occur in the pregnancy and postpartum timeframes accounted for a main portion of the total burden of disease. Women with hypertensive disorders of pregnancy are at significant risk for stroke at a relatively young age. Interval hypertension control and follow-up after pregnancy is likely to be important. Patient education on future stroke issues, exercise and diet counseling could be involved in the postpartum period. Women with a history of hypertensive pregnancy disorders should be candidates for risk modification, including HTN control, lipid management, and weight management.

Our study included a well-established dataset using population-based research with a large sample size, and we explored hypertensive pregnancy disorders as a risk factor for developing stroke. Nevertheless, there are still some limitations of this study. First, although the coding of the NHIRD has not been validated in the recording of all diseases, there were no reports regarding the coding of the severity of stroke or the location of stroke. Data on blood pressure during pregnancy and the control status of hypertensive pregnancy disorders were also unavailable in the database. For these reasons, the effect of hypertensive pregnancy disorders on the severity of stroke or the site of stroke could not be analyzed in detail. Second, the NHIRD registry cannot provide detailed information regarding patients’ laboratory results, lifestyles, past history or family history, and we believe some of these factors, such as smoking, may increase the risk of stroke. Third, given that the population comprises younger females, there might be an underlying disease of the brain that was not noted before, and although the population may be small, we believed that it could also contribute to the increased incidence of stroke [40]. Fourth, there are also some factors that may contribute to stroke, such as autoimmune diseases [41–43], thyroid disease [43–45], oral contraceptive use [46–48], and infectious diseases [49–51], none of which were discussed in this study.

## Conclusions

Our study provided evidence of the increased risk of stroke in patients with hypertensive pregnancy disorders. Compared with those without hypertensive pregnancy disorders, the patients who had experienced such disorders had a 2.134-fold (*P* < 0.001) higher risk of developing stroke in the future. The occurrence of stroke in younger women is an important issue, especially among those who are in the childbearing stage. The results from this study will provide physicians with strong proof of the need for the cautious treatment of patients with hypertensive pregnancy disorders and awareness of possible future stroke problems.

## Supplementary information

**Additional file 1: Table S1.** Distribution of hypertensive pregnancy disorders. **Table S2.** Factors (season, location, urbanization level) associated with stroke using Cox regression. **Table S3.** Sensitivity of factors associated with stroke by using Cox regression.

## Data Availability

The data that support the findings of this study are available from the National Health Insurance Research Database (NHIRD) in Taiwan, but restrictions apply regarding the availability of these data, which were used under license for the current study and thus are not publicly available. The data are, however, available from the authors upon reasonable request and with permission of the National Health Insurance Research Database (NHIRD) in Taiwan.

## Abbreviations

CVA: Cerebrovascular accident
HR: Hazard ratio
JAMA: Journal of the American Medical Association
NHIRD: National Health Insurance Research Database
ICD-9-CM: International Classification of Diseases, Ninth Revision, and Clinical Modification
HTN: Hypertension
USD: United States Dollar
DM: Diabetes mellitus
CKD: Chronic kidney disease
CIs: Confidence intervals
