# Supplementary material for: Association between hypertensive pregnancy disorders and future risk of stroke in Taiwan: a Nationwide population-based retrospective case-control study
Source: BMC Pregnancy Childbirth. 2020 Apr 15;20:217. doi: 10.1186/s12884-020-02898-9 (PMC7160910; doi:10.1186/s12884-020-02898-9)
Supplement: Supplementary file 1 — Additional file 1: Table S1. Distribution of hypertensive pregnancy disorders. Table S2. Factors (season, location, urbanization level) associated with stroke using Cox regression. Table S3. Sensitivity of factors associated with stroke by using Cox regression. [file 12884_2020_2898_MOESM1_ESM.docx]

Table S1. Distribution of hypertensive pregnancy disorders

| **ICD-9-CM** | **Hypertensive Pregnancy Disorders Subgroup** | **n** | **%** |
| --- | --- | --- | --- |
| 642.0 | Hypertension complicating pregnancy, childbirth, and the puerperium | 1,804 | 4.31 |
| 642.1 | Hypertension secondary to renal disease, complicating pregnancy, childbirth, and the puerperium | 121 | 0.29 |
| 642.2 | Other pre-existing hypertension complicating pregnancy | 967 | 2.31 |
| 642.3 | Transient hypertension of pregnancy | 9,255 | 22.10 |
| 642.4 | Mild or unspecified pre-eclampsia | 13,888 | 33.17 |
| 642.5 | Severe pre-eclampsia | 9,957 | 23.78 |
| 642.6 | Eclampsia | 1,103 | 2.63 |
| 642.7 | Pre-eclampsia or eclampsia superimposed on pre-existing hypertension | 1,383 | 3.30 |
| 642.9 | Unspecified hypertension complicating pregnancy, childbirth, or the puerperium | 3,392 | 8.10 |
|  | **Total** | 41,870 |  |

**Table S2.** Factors (season, location, urbanization level) associated with stroke using Cox regression

| **Variables** | **Crude HR** | **95% CI** | **95% CI** | ***P*** | **Adjusted HR** | **95% CI** | **95% CI** | ***P*** |
| --- | --- | --- | --- | --- | --- | --- | --- | --- |
| **Season** |  |  |  |  |  |  |  |  |
| Spring | Reference |  |  |  | Reference |  |  |  |
| Summer | 0.901 | 0.755 | 1.077 | 0.233 | 0.933 | 0.816 | 1.162 | 0.327 |
| Autumn | 0.821 | 0.690 | 0.991 | 0.042 | 1.073 | 0.893 | 1.286 | 0.505 |
| Winter | 0.947 | 0.789 | 1.139 | 0.579 | 0.883 | 0.688 | 1.163 | 0.149 |
| **Location** |  |  |  |  |  |  |  |  |
| Northern Taiwan | Reference |  |  |  | **Multicollinearity with urbanization level** | | | |
| Middle Taiwan | 1.102 | 0.947 | 1.284 | 0.198 | **Multicollinearity with urbanization level** | | | |
| Southern Taiwan | 1.018 | 0.867 | 1.198 | 0.742 | **Multicollinearity with urbanization level** | | | |
| Eastern Taiwan | 1.415 | 1.109 | 1.816 | 0.003 | **Multicollinearity with urbanization level** | | | |
| Outlets islands | 0.611 | 0.153 | 2.455 | 0.482 | **Multicollinearity with urbanization level** | | | |
| **Urbanization level** |  |  |  |  |  |  |  |  |
| 1 (The highest) | 0.937 | 0.811 | 1.063 | 0.446 | 1.367 | 0.680 | 2.139 | 0.248 |
| 2 | 0.801 | 0.630 | 1.026 | 0.101 | 1.170 | 0.730 | 1.775 | 0.175 |
| 3 | 1.003 | 0.826 | 1.218 | 0.872 | 2.001 | 0.701 | 3.370 | 0.347 |
| 4 (The lowest) | Reference |  |  |  | Reference |  |  |  |
| **HR= hazard ratio, CI = confidence interval, adjusted HR= adjusted for variables listed in the table** | | | | | | | | |

**Table S3. Sensitivity of factors associated with stroke by using Cox regression**

| **Hypertensive pregnancy disorders** | **With** | | | **Without** *(Reference)* | | | **Ratio** | **Adjusted HR** | **95% CI** | **95% CI** | ***P*** |
| --- | --- | --- | --- | --- | --- | --- | --- | --- | --- | --- | --- |
| **Sensitivity test** | **Events** | **PYs** | **Rate (per 10^5^ PYs)** | **Events** | **PYs** | **Rate (per 10^5^ PYs)** |  |  |  |  |  |
| **Total** | 351 | 184,427.45 | 190.32 | 904 | 712,815.07 | 126.82 | 1.501 | 2.814 | 2.395 | 3.302 | <0.001 |
| Before delivery was excluded | 221 | 164,509.12 | 134.34 | 744 | 659,712.44 | 112.78 | 1.191 | 2.233 | 1.901 | 2.621 | <0.001 |
| Before and 1 month after delivery were excluded | 201 | 163,241.77 | 123.13 | 703 | 655,012.78 | 107.33 | 1.147 | 2.151 | 1.831 | 2.525 | <0.001 |
| Before and 3 months after delivery were excluded | 198 | 163,208.79 | 121.32 | 698 | 654,982.30 | 106.57 | 1.138 | 2.134 | 1.817 | 2.505 | <0.001 |
| PYs = Person-years; Adjusted HR = Adjusted hazard ratio: Adjusted for the variables listed in Table 3.; CI = confidence interval | | | | | | | | | | | |
